# Diagnosis of an imprinted-gene syndrome by a novel bioinformatics analysis of whole-genome sequences from a family trio

**DOI:** 10.1002/mgg3.107

**Published:** 2014-08-26

**Authors:** Dale L Bodian, Benjamin D Solomon, Alina Khromykh, Dzung C Thach, Ramaswamy K Iyer, Kathleen Link, Robin L Baker, Rajiv Baveja, Joseph G Vockley, John E Niederhuber

**Affiliations:** 1 Inova Translational Medicine Institute, Inova Health SystemFalls Church, Virginia; 2 Department of Pediatric Endocrinology, Inova Children's HospitalFalls Church, Virginia; 3 Fairfax Neonatal Associates PC, Inova Children's HospitalFalls Church, Virginia

**Keywords:** Bioinformatics, *CDKN1C*, diagnosis, exome sequencing, family trio, genomic imprinting, IMAGe syndrome, mutation, rare disease, whole-genome sequencing

## Abstract

Whole-genome sequencing and whole-exome sequencing are becoming more widely applied in clinical medicine to help diagnose rare genetic diseases. Identification of the underlying causative mutations by genome-wide sequencing is greatly facilitated by concurrent analysis of multiple family members, most often the mother–father–proband trio, using bioinformatics pipelines that filter genetic variants by mode of inheritance. However, current pipelines are limited to Mendelian inheritance patterns and do not specifically address disorders caused by mutations in imprinted genes, such as forms of Angelman syndrome and Beckwith–Wiedemann syndrome. Using publicly available tools, we implemented a genetic inheritance search mode to identify imprinted-gene mutations. Application of this search mode to whole-genome sequences from a family trio led to a diagnosis for a proband for whom extensive clinical testing and Mendelian inheritance-based sequence analysis were nondiagnostic. The condition in this patient, IMAGe syndrome, is likely caused by the heterozygous mutation c.832A>G (p.Lys278Glu) in the imprinted gene *CDKN1C*. The genotypes and disease status of six members of the family are consistent with maternal expression of the gene, and allele-biased expression was confirmed by RNA-Seq for the heterozygotes. This analysis demonstrates that an imprinted-gene search mode is a valuable addition to genome sequence analysis pipelines for identifying disease-causative variants.

## Introduction

Whole-genome sequencing (WGS) and whole-exome sequencing (WES) have made tremendous contributions to the diagnosis of rare diseases, contributing both to the discovery of novel disease-causing genes and to the diagnosis of patients with undiagnosed conditions. A recent survey found over 300 publications on the application of WES to rare diseases (Boycott et al. 2013), and the number of patients who undergo genomic sequencing is growing rapidly.

One challenge in the application of genome-wide sequencing to the identification of disease-causing mutations is reducing the set of variants from the millions typically found by WGS to a small number of candidates for expert evaluation (Cooper and Shendure 2011). Commonly applied filtering criteria include rarity in databases of sequences from presumably unaffected individuals, and predicted deleterious impact on the structure, function, or expression of an encoded protein. Filtering by genetic inheritance is another powerful means of prioritizing variants that is becoming more widely adopted as decreasing costs enable sequencing of multiple family members, particularly the mother–father–proband trio. Several bioinformatics pipelines can filter variants by frequency and predicted impact, as well as genetic inheritance (see, e.g., Li et al. 2012; Sincan et al. 2012; Paila et al. 2013; Zhang et al. 2013; Koboldt et al. 2014; Santoni et al. 2014). Most have automated filters for de novo, autosomal dominant, autosomal recessive, and compound heterozygous variants and a few have also implemented searches for variants compatible with X-linked inheritance.

Genomic imprinting is a disease-relevant inheritance process that is not accounted for in current pipelines. Unlike genes that exhibit biallelic expression, imprinted genes are preferentially expressed from one allele in a parent-of-origin specific manner. Manifestation of the disease phenotype associated with a mutation in an imprinted gene depends on the sex of the parent transmitting the mutation. Because an affected child and an unaffected parent can both have the mutation, causative variants in imprinted genes are likely to be excluded by standard family-based pipelines. Two recent studies exemplify the need for imprinted-gene mutation searches. In one study, analysis of WES data based on Mendelian inheritance patterns failed to identify an IMAGe syndrome-causing mutation in the imprinted gene *CDKN1C* (Hamajima et al. 2013), and in the second the imprinted gene *MKRN3* was discoverable as the cause of central precocious puberty because the genotypes and affected status of the particular individuals chosen for sequencing coincidentally matched an autosomal dominant pattern (Abreu et al. 2013).

In the present study, we used family trio-based WGS to diagnose a child with a rare condition for which multiple levels of investigation did not reveal an underlying explanation. When application of the standard genetic inheritance filters failed to identify a likely causative mutation, we designed a new filtering strategy specific for the analysis of imprinted-gene variants and implemented this approach using publicly available tools. This novel analysis pipeline identified a single mutation, NM_000076.2:c.832A>G (p.Lys278Glu) in the imprinted gene *CDKN1C* (OMIM 600856), and provided the diagnosis of IMAGe syndrome for the proband. The imprinted-gene filters can be applied to the identification of causative mutations for other imprinted-gene diseases, and may thus provide molecular diagnoses for some individuals for whom standard family-based pipelines have not revealed an etiology.

## Materials and Methods

### Ethics statement

This study was approved by the Inova Research Center and Western Institutional Review Boards (WIRB #20121680). Full informed consent was obtained from all adult study participants and from the parents of minors.

### Sample collection and processing

Whole blood was collected from all participants in BD vacutainer or microtainer (for infants) potassium (K2) EDTA tubes and sent to the ITMI Core Laboratory. At the laboratory, whole blood was centrifuged and fractionated into packed cells and plasma. Genomic DNA (gDNA) was isolated from the packed cell fraction using a Qiagen (Germantown, MD) DNA Midi kit on a QiaSymphony DNA isolation platform. DNA quantification and QC were performed by spectrophotometry on a NanoDrop 8000 instrument (Thermoscientific, Wilmington, DE), and by fluorimetry using PicoGreen or Quantiflour dyes on a TECAN F200 instrument (TECAN US, INC, Morrisville, NC). Additionally, qualitative assessment of gDNA was performed by agarose gel electrophoresis.

### Whole-genome sequencing

High-quality genomic DNA (3 *μ*g) was sent to the Illumina FastTrack Services (FTS) laboratory (San Diego, CA) for WGS. Prior to sequencing, DNA quality was checked by microarray analysis on the HumanOmni2.5-8 array, using Infinium chemistry. WGS was performed using protocols recommended by the kit and instrumentation manufacturer(s) as described below. Briefly, paired-end libraries were manually generated from 500 ng to 1 *μ*g of gDNA using the Illumina TruSeq DNA Sample Preparation Kit. The quality of the libraries was assessed by gel electrophoresis, or chip electrophoresis on the Agilent BioAnalyzer (Agilent Technologies, Santa Clara, CA), using the Agilent DNA 1000 chip. Libraries were quantified by qPCR as described in the Illumina Sequencing Library qPCR Quantification protocol. DNA libraries were denatured, diluted, and clustered onto v3 flow cells, using the Illumina TruSeq Cluster Kit v3 and the Illumina cBot™ system. Clustered v3 flow cells were loaded onto HiSeq 2000 instruments and sequenced on 100 base pair (bp) paired-end, nonindexed runs, using Illumina TruSeq SBS v3 Reagents. Illumina HiSeq Control Software (HCS) and real-time analysis (RTA) were used on HiSeq 2000 sequencing runs for real-time image analysis and base calling.

Read alignment to human reference assembly hg19 (Lander et al. 2001) and variant calling were conducted by the Illumina Whole Human Genome Sequencing Service Informatics Pipeline version 2.01-02 using the iSAAC package (Raczy et al. 2013).

### Variant filtering

Single-nucleotide polymorphisms (SNPs) and small insertions/deletions (indels) with “PASS” filters and quality scores of at least 30 in mother, father, and infant genomes were extracted using gVCFtools v0.16 (https://sites.google.com/site/gvcftools/) and bcftools version 0.1.19-44428cd (Li et al. 2009), then annotated with snpEff v3.3 (Cingolani et al. 2012), and loaded into a GEMINI version 0.6.4 relational database (Paila et al. 2013).

Variants were further filtered using GEMINI query tools for de novo, recessive, and compound heterozygous mutations adapted for the following criteria. Variants were required to (1) have a minimum read depth of 10, (2) be fully called in the trio, and (3) have possibly deleterious effects, defined as having impact severity of high or medium as calculated by snpEff (Cingolani et al. 2012) or as being annotated as pathogenic or probably pathogenic in ClinVar (Landrum et al. 2014) or disease-causing in HGMD. Causative variants were hypothesized to be very rare and to have frequencies in the NHLBI GO Exome Sequencing Project ESP6500 dataset (http://evs.gs.washington.edu/EVS/) and the 1000 Genomes Project Consortium (2012) data of <0.5% for de novo mutations and <1% for autosomal recessive and compound heterozygous mutations. Variants present in our in-house WGS database of 659 mother–father–newborn trios (ascertained as cases or controls for our preterm birth study) with inheritance consistent with the proband's family were also excluded. Since not all variants can be phased, we only excluded candidate pairs of variants from the compound heterozygotes search that were clearly incompatible with this inheritance pattern. Candidate variant pairs were excluded if both variants were unambiguously inherited from the same parent, or if either variant was homozygous in a parent.

To identify candidate causative variants in imprinted genes, an imprinted-gene variant search mode was implemented using the GEMINI query language. In addition to satisfying the frequency and deleterious effect criteria above, the imprinted-gene variants were required to lie in a gene known to undergo genomic imprinting, and to exhibit inheritance consistent with the gene's parent-of-origin expression. We reasoned that, in a family with an affected child with a mutation in a maternally expressed gene, the child would be either heterozygous or homozygous for the mutation, and the child's unaffected, carrier mother would be heterozygous. The unaffected father would be either homozygous reference, or heterozygous if he inherited the mutation from his own father. Analogous criteria were implemented for paternally expressed genes.

All variants resulting from the automated analyses were evaluated by clinical genetics experts for congruence between the known function of the gene and previously reported disease associations and the proband's phenotype.

### Pathogenicity annotation

Pathogenicity annotations were obtained from two sources, ClinVar and HGMD. The ClinVar 12/30/13 VCF file (Landrum et al. 2014) was downloaded from NCBI. After splitting multiallelic loci, variants were left aligned and trimmed with GATK version 2.8.1 (McKenna et al. 2010). Additional annotations were obtained from the HGMD Professional version 2013.2 VCF file (BIOBASE). The data were uploaded as GEMINI database annotations following the recommended steps (http://gemini.readthedocs.org/en/latest/index.html).

### Imprinted regions

A list of human imprinted genes and their parent-of-origin expression was downloaded from the Catalog of Parent of Origin Effects, January 2011 version (Morison et al. 2001). Separate lists were maintained for maternally expressed and paternally expressed genes. Genes with ambiguous or unknown expression were assigned to both lists. Genomic coordinates for the imprinted genes were obtained from the UCSC genome browser database tables (Meyer et al. 2013).

### Variant confirmation and return of results

Variants were confirmed via traditional bidirectional Sanger sequencing using the original research samples. Gene-specific forward and reverse primer sequences flanking the nucleotide of interest on the *CDKN1C* gene were designed using the Primer3 program (http://biotools.umassmed.edu/bioapps/primer3_www.cgi), and checked by Primer-BLAST (http://www.ncbi.nlm.nih.gov/tools/primer-blast/). M13 forward and reverse sequences were added to the 5′ ends of these gene-specific sequences to obtain the forward and reverse primers, M13F-CDKN1C-f1 (5′-TGTAAAACGACGGCCAGTCGACGTAAACAAAGCTGACC-3′) and M13R-CDKN1C-r1 (5′-CAGGAAACAGCTATGACCGCTGTACTCACTTGGCTCACC-3′). The gene-specific sequences in the forward and reverse primers correspond to genomic coordinates 2,884,287–2,884,268 and 2,884,008–2,883,988 on NC_000011.10, the Homo sapiens chromosome 11, GRCh38 primary assembly. Primers were purchased from Eurofins MWG Operon/Fisher Scientific (Huntsville, AL).

The 336-bp (including primers) target region was amplified using 200 nmol/L of each primer, 50 ng of genomic DNA, the FailSafe™ PCR system (Epicentre/Illumina, Madison, WI), and PCR System Premix I, and the following touchdown PCR conditions: (95°C for 5 min) × 1 cycle; (95°C for 30 sec, touchdown from 65°C to 55°C [−0.5°C/cycle] for 30 sec, 72°C for 30 sec) × 20 cycles; (95°C for 30 sec, 55°C for 30 sec, 72°C for 30 sec) × 20 cycles; (72°C for 7 min) × 1; and finally infinite hold at 4°C, until further processing. PCR amplicons were purified using the AgenCourt AMPure XP PCR Purification system (Beckman Coulter, Brea, CA).

Purified product was bidirectionally sequenced using the BigDye v3.1 fluorescent dideoxy terminator kit (Applied BioSystems, Grand Island, NY), and M13 forward (5′-TGTAAAACGACGGCCAGT-3′) and reverse (5′-CAGGAAACAGCTATGACC-3′). The chromatograms obtained were analyzed using DNA Baser Sequence Assembler version 4.12 (Plimus, Campbell, CA).

Results were returned to the participants through individual counseling sessions following confirmatory sequencing by a CLIA lab (GeneDx, Gaithersburg, MD) using independently obtained buccal swap samples. The confirmed variant was deposited in ClinVar (http://www.ncbi.nlm.nih.gov/clinvar/).

### RNA extraction

Whole blood samples, collected in PAXgene blood stabilization tubes (Fisher Scientific, Pittsburgh, PA), were extracted for total RNA. The methods were adapted from the MagMax for Stabilized Blood Tubes RNA Isolation Kit (Life Technologies, Grand Island, NY) with automation on a Freedom EVO150 liquid handling robot (Tecan US, Inc., Morrisville, NC). The samples were centrifuged to remove the stabilization buffer, digested with Proteinase K, and filtered using a TurboFilter96 plate (Qiagen) to produce a RNA-rich supernatant. On the liquid handling robot, magnetic beads bind the nucleic acids, DNase incubation digests the DNA, and RNA is eluted. The RNA was concentrated for downstream processes using the ZR-96 RNA Clean & Concentrator kit (Zymo Research Corporation, Irvine, CA). RNA samples were sent in liquid nitrogen to Expression Analysis, Inc. (Durham, NC) for library construction and RNA-Seq.

### Library construction for RNA-Seq

#### Globin clear

Globin mRNA was substantially depleted from total RNA samples using the GlobinClear-Human Kit (Life Technologies), essentially as described by the vendor. Briefly, 1.25 *μ*g of total RNA isolated from whole blood was combined with biotinylated capture oligonucleotides complementary to globin mRNAs and the mixture was incubated at 50°C for 15 min to allow duplex formation. Streptavidin magnetic beads are added to each specimen, and the resulting mixture was incubated for an additional 30 min at 50°C to allow binding of the biotin moieties by Streptavidin. These complexes, comprising Streptavidin magnetic beads bound to biotinylated oligonucleotides that are specifically hybridized to globin mRNAs, were then captured using a magnet. The globin-depleted supernatant was transferred to a new container and further purified using RNA-binding beads. The final globin mRNA-depleted RNA samples were quantitated by spectrophotometry using a NanoDrop ND-8000 spectrophotometer.

#### RNA-seq – TruSeq stranded mRNA

Globin-depleted total RNA samples were converted into cDNA libraries using the TruSeq Stranded mRNA Sample Prep Kit (Illumina). Starting with 100 ng of total RNA, polyadenylated RNA (primarily mRNA) was selected and purified using oligo-dT-conjugated magnetic beads. This mRNA was chemically fragmented and converted into single-stranded cDNA using reverse transcriptase and random hexamer primers, with the addition of Actinomycin D to suppress DNA-dependent synthesis of the second strand. Double-stranded cDNA was created by removing the RNA template and synthesizing the second strand in the presence of dUTP (deoxyribouridine triphosphate) in place of dTTP (deoxythymidine triphosphate). A single A base was added to the 3′ end to facilitate ligation of sequencing adapters, which contain a single T base overhang. Adapter-ligated cDNA was amplified by polymerase chain reaction to increase the amount of sequence-ready library. During this amplification the polymerase stalls when it encounters a U base, rendering the second strand a poor template. Accordingly, amplified material used the first strand as a template, thereby preserving the strand information. Final cDNA libraries were analyzed for size distribution and using an Agilent Bioanalyzer (DNA 1000 kit; Agilent), quantitated by qPCR (Kapa Library Quant Kit; Kapa Biosystems, Wilmington, MA), then normalized to 2 nmol/L in preparation for sequencing.

### RNA-Seq

Samples were assessed for quality via Agilent Tapestation, followed by library quantification by qPCR. Libraries were then normalized to 2 nmol/L and then pooled in equimolar amounts. The pooled libraries were denatured using fresh 0.1 N NaOH and diluted to a final loading concentration of 14 pmol/L. For sequencing runs on the HiSeq 2000, pools were placed on an Illumina cBot (v1.5.12.0) for cluster generation. For HiSeq 2500 runs, on-board flowcell clustering was used to generate clusters on the sequencer. Templates were attached to the flowcell via a dense lawn of oligonucleotides that bind to the sequencing adapters added during sample preparation, which are extended and then denatured. The flowcells were then sequenced through 50 bases, paired end, with an eight-base index cycle on either an Illumina HiSeq 2000 (HiSeq Control Software v1.5.15.1) or an Illumina HiSeq 2500 (HiSeq Control Software v2.0.12.0). All libraries were sequenced using Illumina v3 sequencing reagents on the HiSeq 2000 and v1 rapid sequencing reagents on the HiSeq 2500.

Upon completion of sequencing, basecall files were converted into FASTQ files using Illumina Software (CASAVA). To prepare the reads for alignment, the sequencing adapters and other low-quality bases were clipped (Aronesty 2013). Reads were aligned to External RNA Controls Consortium (ERCC) sequences to assess the success of library construction and sequencing. A subset of the reads (∼1 million reads) were aligned to other spiked-in control sequences (PhiX and other Illumina controls used during library preparation), residual sequences (globin and ribosomal RNA), and poly-A/T sequences that persisted after clipping. The reads were also aligned to a sampling of intergenic regions to assess the level of DNA contamination level. RSEM v1.2.0 (Li and Dewey 2011) was used to quantify genes and transcripts using the UCSC knownGene transcriptome (Meyer et al. 2013). Reads that remained unaligned to the transcriptome may be due to misannotation, lack of annotation, or be from genomic DNA. To determine the origin of all reads as a method of quality control, the unaligned reads were aligned to the full genome (not transcriptome) using BWA 0.6.2 (Li and Durbin 2009). RNA-Seq quality metrics are listed in Table S1.

## Results

### Patient description

The patient was an ex-34-week-old Caucasian male born to a mother with prenatally diagnosed Factor II (c.20210G>A) and *MTHFR* (c.677C>T) heterozygosity. Prenatal nuchal and first trimester serum screening showing increased risk of Down syndrome (1:72), prompting chorionic villus sampling, which showed a normal male chromosome complement (46,XY). Subsequent prenatal examinations showed evidence of severe intrauterine growth restriction (IUGR) and oligohydramnios, and the infant was delivered by cesarean section at 34 3/7 weeks gestation because of these concerns. The birthweight was 1160 g (>2 SD below the mean for gestational age, ∼50th centile for a 28-week gestation). After delivery, in summary, the infant was found throughout his ∼7 week NICU hospitalization to have hypoaldosteronism, but without signs of glucocorticoid deficiency, and with no adrenal anomalies identified by ultrasound. In the second week of life, he had an aldosterone/plasma renin activity ratio of 0.0 (normal 0.9–28.9), with aldosterone level <1 ng/dL (normal 2–70 ng/dL) and an elevated plasma renin activity of 207.52 ng/mL per h (normal 0.25–5.82 ng/mL per h). He was documented to have hyponatremia and hyperkalemia, though with no frank adrenal crises – morning cortisol level was normal at 3.7 *μ*g/dL (normal 3.7–19.4 *μ*g/dL); ACTH stimulation test was not performed during the NICU admission. Additional findings included hypercalciuria, grade II hydronephrosis, small atrial septal defect and patent ductus arteriosus, and sagittal craniosynostosis, the latter of which required an initial surgery in infancy, as well as a revision in the second year of life. Physical examination by a clinical geneticist during the NICU stay revealed frontal bossing, ear dysplasia (overfolded, simple ear), scrotal hypoplasia, and bilateral mild cutaneous 2–3 toe syndactyly. Consanguinity was denied, and family history was noncontributory, with no other similarly affected individuals known (however, once the molecular diagnosis was achieved, a relative of the maternal grandfather who died in infancy of unclear reasons was recalled). Per clinical genetics evaluation, there was felt to be no obvious known syndrome or underlying disorder. Genetic and other etiologic-based testing during the NICU hospitalization, none of which revealed an explanation, included: karyotype (done prenatally due to concern for IUGR), high-density oligonucleotide/SNP microarray, biochemical testing for Smith–Lemli–Opitz syndrome through 7-dehydroxycholesterol level, a TORCH panel, and Russell–Silver syndrome testing (including H19 methylation and uniparental disomy for chromosome 7). After NICU discharge, he was managed for aldosterone deficiency with fludrocortisone and salt replacement, with regular monitoring. As the molecular diagnosis was achieved only a few days before the repeat craniosynostosis surgery, stress-dose steroids were recommended for this procedure as well as for other clinical situations necessitating this intervention.

### Whole-genome sequence analysis

**Figure 1:**
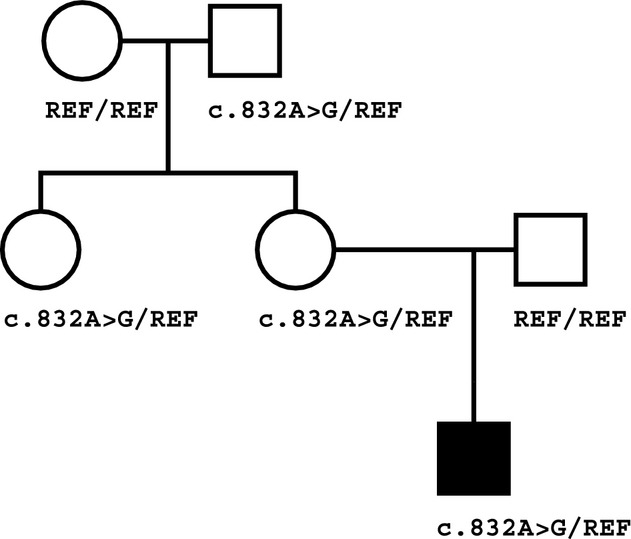
Family pedigree. Squares: males; circles: females; black: clinically affected. Individuals are labeled by genotype at position *CDKN1C* c.832, with the reference allele (without the mutation) indicated as “REF.”

Since the proband's condition remained unexplained after extensive clinical testing, we performed family-based WGS in an attempt to identify a causative mutation. DNA was isolated from peripheral blood collected from the proband and both parents. Average genomic coverage was >40× for each family member (Table1). The 5,077,401 variants in the trio were filtered for genotype quality, likelihood of being disease causative, and inheritance mode. Given the apparently sporadic disease (Fig.1), we hypothesized that the causative mutation would be de novo, but no obvious genetic explanation consistent with a de novo mutation was found. The single variant passing the filtering criteria, chr22:21570310 G>C, falls in a predicted alternate transcript 6538 base pairs upstream of the *GGT2* gene, and was rated by clinical genetics experts as unlikely to account for the majority of the proband's symptoms.

**Table 1:** Whole-genome sequencing statistics for the trio

Family member	Yield (Gb)^1^	Average coverage	SNPs	Indels	SNP TiTv	SNP Het/Hom
Proband	132.03	45.08x	3,506,132	384,236	2.08	1.62
Mother	118.94	40.04x	3,563,863	388,572	2.08	1.66
Father	123.39	42.34x	3,499,397	380,916	2.08	1.63

Gb, gigabases; Indels, insertions and deletions; SNPs, single-nucleotide polymorphisms.

1 Passing filter and aligned.

Searches for autosomal recessive and compound heterozygous mutations also produced no likely candidates. The four genes with putative homozygous mutations, *FAM86B1*,*MUC20*,*MUC4*, and *TSEN54*, and one gene with putative compound heterozygous mutations, *MUC19*, lie in genomic regions difficult to sequence by next-generation sequencing technology and/or are common hits in searches for recessive variants in families with unrelated conditions in our in-house database. Diseases associated with the remaining genes with compound heterozygous variants, *HSPG2*,*ZFAT*,*TLR1*, and *SYNE1*, are not consistent with the phenotype of the proband.

### Imprinted-gene analysis

Since modeling the Mendelian modes of inheritance compatible with the family history did not identify a likely pathogenic mutation, and since Russell–Silver syndrome had been considered as a clinical diagnosis, we decided to test whether the proband's phenotype resulted from a mutation in an imprinted gene. We developed a novel bioinformatics analysis to search for imprinted-gene mutations that are overlooked by Mendelian inheritance-based search strategies. This analysis revealed a single variant with inheritance consistent with the known parent-of-origin expression of the affected gene, and which passed quality, population frequency, and deleterious effect filters. This variant is a heterozygous mutation in the imprinted gene *CDKN1C*, chromosome 11 g.2905353T>C (hg19), corresponding to NM_000076.2:c.832A>G (p.Lys278Glu), recently reported to cause IMAGe syndrome (the acronym stands for Intrauterine growth restriction, Metaphyseal dysplasia, Adrenal hypoplasia congenita, and Genital anomalies) (Arboleda et al. 2012). On review of the literature, the patient's phenotype was felt to be highly congruent with those of previously reported patients, both with the specific mutation as well as with other IMAGe syndrome-causing mutations in the same gene, including the affected organ systems and specific clinical manifestations (Arboleda et al. 2012). As confirmed by Sanger sequencing (Fig. S1), the proband and his mother are heterozygous for the *CDKN1C* mutation while his father is homozygous reference (Fig.1). This suggests that the proband inherited the mutation from his mother, consistent with the documented maternal expression of this gene.

If this mutation is the causative mutation for the proband's symptoms, the unaffected mother would most likely carry the mutation on her paternal allele. To test this possibility, we analyzed WGS data from the proband's maternal grandparents, both clinically unaffected. The *CDKN1C* mutation was detected in the grandfather but not the grandmother, a finding confirmed by Sanger sequencing (Fig. S1). The proband's unaffected maternal aunt was also tested and found to carry the mutation. Thus, the inheritance pattern observed in this family (Fig.1) is consistent with maternal-specific expression of this gene.

### Confirmation of parent-of-origin expression

Allele-biased expression of the *CDKN1C* mutation was analyzed by RNA-Seq (Table2). Although the total number of reads at this position is too low for a statistically significant result, the data are consistent with the variant allele being relatively highly expressed in the proband compared to his mother, grandfather, and aunt. Comparing the family members, the read counts are suggestive of both reduced mRNA expression of the paternal allele, as well as higher relative expression of the allele with the mutation in the affected individual. The RNA-Seq data further confirm the presence of the mutation in the proband, mother, aunt, and grandfather. The detection of some reads from the paternal allele is consistent with the leaky and variable expression of this gene previously observed in primary lymphocytes (Algar et al. 2000).

**Table 2:** Relative expression by RNA-Seq of the chromosome 11 g.2905353T>C (hg19) alleles, corresponding to NM_000076.2:c.832A>G

Family member	Number of reads			
	Total	Reference (T)	Variant (C)	% Variant (C/T)
Proband	8	3	4	57.1
Mother	20	16	4	20.0
Father	29	29	0	0.0
Grandmother	16	16	0	0.0
Grandfather	8	6	2	25.0
Aunt	33	26	7	21.2

## Discussion

Although WGS/WES is increasingly used to assist in the diagnosis of rare disorders, a probable genetic cause is detected in only ∼25% of cases (Jacob et al. 2013; Yang et al. 2013). There are many reasons why sequencing for diagnosis can be unsuccessful, including a nongenetic etiology, or a mutation in a region not well characterized by genome-wide sequencing technologies (Yang et al. 2013). In family-based analyses, mutations may also be overlooked by applying an inappropriate inheritance model. Since currently available tools that filter by genetic inheritance are limited to Mendelian inheritance modes, diseases caused by mutation of an imprinted gene are more likely to be undiagnosed by family-based analysis pipelines. We addressed this gap by implementing a search customized for imprinted-gene variant analysis, thereby providing a diagnosis for the proband when both extensive clinical testing and standard WGS pipelines were nondiagnostic.

The analysis of imprinted-gene variants was limited to known imprinted protein-coding genes. Identification of imprinted genomic regions is an area of active research that may identify genes not previously known to be imprinted, or which are erroneously labeled as imprinted (Lawson et al. 2013). Since the imprinted-gene filter was implemented using genomic coordinates, it is easily extensible to other gene lists, as well as to other genomic regions, including nonprotein-coding genes and regulatory regions.

We implemented the imprinted-gene variant search by adapting publicly available tools and databases. The genetic inheritance filtering was implemented with the GEMINI software package (Paila et al. 2013), since the flexibility of its query language and the freely available source code facilitated the development of custom queries. Imprinted-gene variant searches can also be implemented in other software packages that can interrogate variants based on inheritance patterns. Our proof-of-principle search strategy focused on the family trio, and analogous approaches can be applied to the identification of variants in imprinted genes in other family groups.

Since the proband described here carries a previously reported disease-associated variant, the diagnosis could have been achieved by searching for known pathogenic mutations. However, for family-based analyses, accounting for maternal expression of the impacted gene is necessary to avoid exclusion of the variant due to its presence in unaffected family members. Imprinting-aware filtering for known pathogenic variants could be accomplished by extending existing mutation databases to include parent-of-origin inheritance information routinely and accurately. Alternatively, an imprinted-region filtering strategy such as that described here could be employed, and could identify both known and novel pathogenic variants.

Proof of causality is the rate-limiting step in diagnosis by WGS/WES. Currently, the most convincing method is identification of the same rare variant in unrelated individuals with the same phenotype. The *CDKN1C* mutation c.832A>G (p.Lys278Glu) found in the proband described here was previously identified in one unrelated individual with IMAGe syndrome (Arboleda et al. 2012). As the second reported occurrence of this variant in a patient with the IMAGe syndrome phenotype, our results provide corroborating evidence that the mutation is causative for this disorder.

Although the patient's clinical manifestations (which include IUGR, adrenal deficiency, hypercalciuria, a subtle but characteristic facial phenotype, craniosysnostosis, and genital features) match very closely with previously reported cases, the adrenal phenotype appears to be subtly different: he did not suffer neonatal onset adrenal crises and specifically had evidence for mineralocorticoid but not glucocorticoid deficiency. This suggests a minor but clinically important expansion of the reported phenotype. Although we recommended stress-dose steroids in the future, it is important to note that he fared well during his initial surgery without this intervention.

The WGS analysis reported here was employed in a “hypothesis-free” manner using variant properties and inheritance data to prioritize variants. Bioinformatics analysis initially identified the *CDKN1C* mutation without any filtering based on the clinical phenotype. However, clinical consideration remains important in order to help determine whether the genotype–phenotype data correlate appropriately. Here, the clinical information was highly supportive of the mutation being responsible for the patient's phenotype. This clinical consideration is especially important because the evidence for many mutations causing disease is unclear, even in some relatively well-described Mendelian disorders (Piton et al. 2013).

It is anticipated that WGS/WES will play an increasing role in the diagnosis of rare diseases. The success of the analysis described here, and examples of the application of inappropriate genetic inheritance models to the identification of disease-causative mutations in imprinted genes (Abreu et al. 2013; Hamajima et al. 2013), suggest that family-based analyses pipelines should be expanded to include imprinted-gene variant analysis, particularly for cases for which Mendelian inheritance filters fail to identify plausible candidates. Implementation of such a strategy will contribute to increasing the rate of successful diagnoses achieved through application of WGS/WES technology.

## Supporting Information

Table S1 RNA-Seq quality metrics.

Figure S1 Sanger sequencing results. The proband, mother, and maternal grandfather are heterozygous for the mutation *CDKN1C* c.832A>G and the father and maternal grandmother are homozygous reference.
