# Supplementary material for: Diagnosis of an imprinted-gene syndrome by a novel bioinformatics analysis of whole-genome sequences from a family trio
Source: Mol Genet Genomic Med. 2014 Aug 26;2(6):530–8. doi: 10.1002/mgg3.107 (PMC4303223; doi:10.1002/mgg3.107)
Supplement: Figure S1 — Sanger sequencing results. The proband, mother, and maternal grandfather are heterozygous for the mutation CDKN1C c.832A>G and the father and maternal grandmother are homozygous reference. [file mgg30002-0530-sd2.pdf]

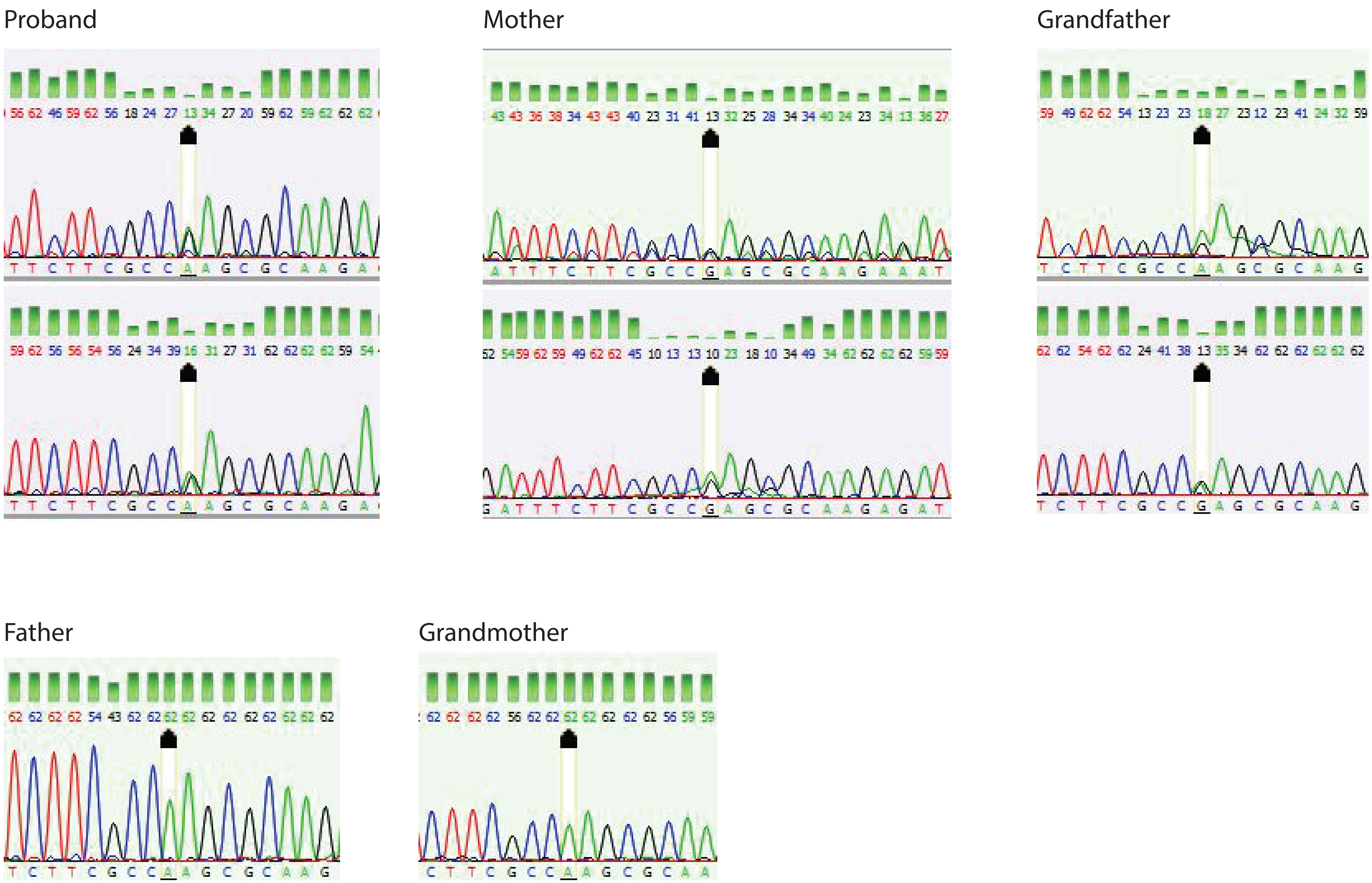

Supplementary Figure S-1. Sanger sequencing results. The proband, mother, and maternal grandfather are heterozygous for the mutation *CDKN1C* c.832A>G and the father and maternal grandmother are homozygous reference.
